# A peek at the other side of the coin: Tumor‐suppressor role of microRNAs expressed by pancreatic cancer‐associated fibroblasts

**DOI:** 10.1002/ctm2.1357

**Published:** 2023-08-04

**Authors:** Gerik Tushoski‐Alemán, Aaron Davidson, Kelly Herremans, Chris Forsmark, Weizhou Zhang, Steven Hughes, Song Han

**Affiliations:** ^1^ Department of Surgery College of Medicine University of Florida Gainesville Florida USA; ^2^ Department of Medicine College of Medicine University of Florida Gainesville Florida USA; ^3^ Department of Pathology Immunology and Laboratory Medicine College of Medicine University of Florida Gainesville Florida USA

To the Editor:

One of the most distinguishable characteristics of pancreatic ductal adenocarcinoma (PDAC) is its extensive desmoplastic stroma, and alpha smooth muscle actin (αSMA)‐expressing cancer‐associated fibroblasts (CAFs) that represent the largest component of this tumor‐associated stroma (TAS). It is well established that reciprocal cancer‐stroma cell interactions within the PDAC microenvironment influence cancer progression, response to chemotherapy, and immune tolerance. microRNAs (miRNAs) are small non‐coding RNAs that contribute to the regulation of gene expression. Their biological impact on cancers can be both oncogenic (onco‐miR) or tumor suppressive (TS‐miR).^1^ miRNAs transported by extracellular vesicles (EVs) are effective communicators in tumor microenvironment (TME), and miRNA‐RNA networks influence core PDAC signaling pathways during tumorigenesis. By and large, CAF‐derived miRNAs are perceived as oncogenic (onco‐miR), contributing to chemoresistance, metastasis, and progression. In contrast, our group reported that CAF‐derived miR‐145‐5p, efficiently transferred to adjacent PDAC cells, inhibits growth and induces apoptosis in cultured PDAC cells.^2^ Others have recently reported similar findings; CAFs dynamically express tumor‐suppressive miRNA in various cancers.^3^ This inspired us to reinvestigate the role of CAF‐derived miRNAs in greater depth.

We previously identified a set of miRNAs specifically and abundantly expressed in PDAC‐derived primary CAFs using nanoCount miRNA profiling (nanoString).^4^ Here, we focused on an in silico analysis on a set of miRNAs differentially expressed by pancreatic CAFs (deCAF‐miRNAs) and explored the other side of the coin—their putative tumor‐suppressive proprieties. Starting with the most abundantly expressed deCAF‐miRNA, we first performed a thorough, systematic literature search on the Medline database using the PubMed search engine and excluded those that have less than 20 reported studies. Ingenuity pathway analysis (IPA) further defined a final set of six deCAF‐miRNAs specifically involved in pancreatic adenocarcinoma signaling (see Document S1 for methods). These are miR‐145‐5p and miR‐199a‐5p, followed by miR‐136‐5p, miR‐137‐3p, miR‐127‐3p, and miR‐139‐3p (in order of expression abundance, Table S1). To our surprise, an overwhelming number of research articles (95.2%, 550/578) described this set of deCAF‐miRNAs as tumor‐suppressive, whereas only 4.8% (28/578) of studies supported a variety of oncogenic properties across cancer types (Figure 1A and Table S1). This included the reported function of downregulating tumor progression (inhibited cellular proliferation, activated apoptosis, and increased chemosensitivity, etc.) in the in vitro studies, and reduced tumor growth and/or increased survival in the in vivo studies. Noticeably, the top two most abundant deCAF‐miRNAs, that is, miR‐145‐5p and miR‐199a‐5p, were primarily tumor suppressive, documented in 97.7% (339/347) and 89.5% (111/124) articles, respectively. These data indicate a strong potency of the antitumor property of the set of deCAF‐miRNAs. Alas, scarce data are obtainable regarding these miRNAs or their impact on PDAC specifically. Yet miR‐145‐5p (11 studies), miR‐137‐3p (five studies), and miR‐139‐3p (one study) have in consonance shown their antitumor function in PDAC (Table S1). Given the finite number of reports, the data clearly warrant further investigation to better understand the actual role of CAF‐derived miRNAs.

**FIGURE 1:**
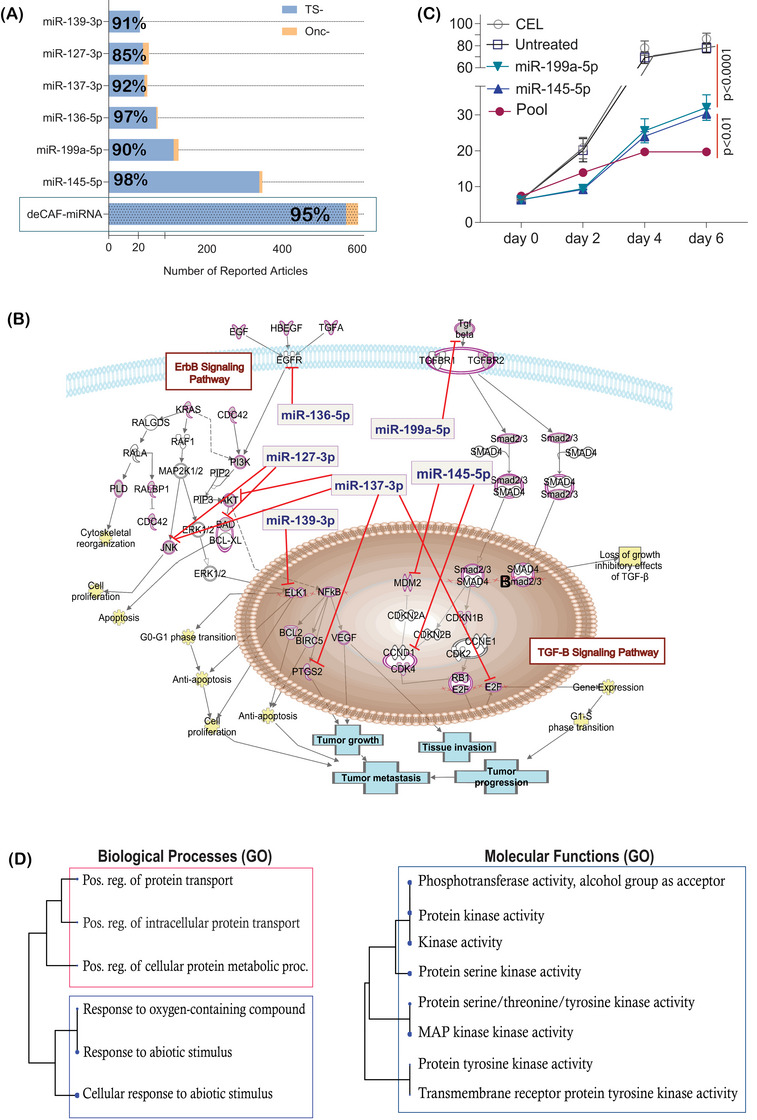
(A) Bar chart displays the percentile of studies that reported the TS‐function (blue colored) and oncogenic function (orange colored) of each differentially expressed miRNAs in cancer‐associated fibroblasts (deCAF‐miRNAs). Actual numbers of literature articles are aligned with *x*‐axis; (B) The miRNA‐mRNA network implicating the set of deCAF‐miRNAs and their target genes in core pancreatic ductal adenocarcinoma (PDAC) signaling pathways generated through ingenuity pathway analysis (IPA) prediction and KEGG annotation. Colored nodes represent target molecules, and solid lines connect their relationships with upstream miRNAs; (C) Cell viability and proliferation curve were measured by alamarBlue assay (Thermo Scientific). The *y*‐axis has arbitrary units of fluorescent readings measured by Clariostar plate reader. Exogenous transfection of 1 nM mimics of miR‐145‐5p, miR‐199a‐5p, or the pool of both into PDX‐derived human primary PDAC cell line PC‐1. Untreated is non‐transfection control and CEL is mock transfection control of *Caenorhabditis elegans* (Cel‐miR‐39‐3p); (D) Top most significant (sorted by FDR) pathways in gene ontology (GO) terms of biological processes (left) and molecular functions (right) arranged in a hierarchical clustering tree.

miRNA regulation forges miRNA‐RNA networks that heavily converge on tumorigenesis signaling pathways. Thus, we next asked to what extent this set of defined deCAF‐miRNAs influences the PDAC core pathways. miRNA target filter identified a total of 11 mRNA targets from the six deCAF‐miRNAs (within experimental evidence or high confidence prediction) functionally relevant to pancreatic adenocarcinoma signaling pathways (Figure 1B and Table S2). Interestingly, the top two deCAF‐miRNAs appear in liaison, targeting the TGF‐β pathway: with miR‐199a‐5p inhibiting the master regulator TGF‐β and miR‐145‐5p downregulating the non‐canonical downstream cascades of CDK4 and MDM2 (Figure 1B). We also demonstrated in primary cell culture that exogenously enhanced expression of both miR‐145‐5p and miR‐199a‐5p by mimic transfection, either individually or in combination, inhibits PDAC cell growth (Figure 1C). Meanwhile, a team of the remaining four deCAF‐miRNAs appears to target ERBB/EGFR signaling, another frequently dysregulated PDAC pathway. This occurs through upstream EGFR inhibition by miR‐136‐5p, and downstream PI3K/AKT pathway dysregulation by miR‐137‐3p and miR‐127‐3p, as well as MAPK substrates of ELK1 inhibition by miR‐139‐3p. Finally, the two core PDAC signaling pathways are also intertwined with one another in non‐canonical fashion (i.e., TGF‐β is also known to be capable of activating signal proteins in the RAS/MAPK or PI3K/AKT pathways). Gene ontology (GO) annotations further highlight the focal points of target genes in protein secretion processes (e.g., cytokines) under abiotic stimulation (biological process) and in phosphorylation catalysis, including MAPK substrates (molecular function) (Figure 1D).

CAFs are phenotypically and functionally at least dichotomous by spatial distribution and during tumorigenesis. Current theory postulates that CAF subtypes are responsible for this double‐sided function. For example, myofibroblastic (αSMA^high^ myCAFs) are tumor restraining, and inflammatory (αSMA^low^/IL6^high^ iCAFs) and antigen‐presenting (MHCII^+^ apCAFs) are immunosuppressive.^5^ Recently, a TGF‐β co‐receptor CD105 demarcated two sub‐CAF populations identifying CD105^+^ CAFs as tumor permissive, whereas CD105^−^ CAFs are tumor suppressive.^6^ While the mechanism(s) of these paradoxical roles of CAFs remains unclear, efforts continue to stratify stromal subtypes have all been based on transcriptomic analyses.^7^ What roles and how paramount regulatory miRNAs in the CAFs remain unexplored. New information on converging paths of CAF‐derived miRNAs and their targets may prove very important in defining CAF subtypes and creating a consensus for CAF nomenclature, a current challenge in CAF heterogeneity studies.

The influence of CAFs in PDAC signaling is complex, intertwined, and dynamic. TGF‐β signaling plays a dual role of tumor suppressor in the early stages of PDAC, and pro‐oncogenic and pro‐metastatic as the tumor progresses. It also transduces PI3/Akt and MAPK/JNK pathways through a non‐canonical SMAD‐independent pathway.^8^ TGF‐β signaling is actively involved in CAF formation as well as plasticity. The myCAF phenotype is highly TGF‐β driven, and experimentally TGF‐β inhibits iCAF formation—the bad sibling.^9^ This plasticity may present a therapeutic opportunity for a “stroma‐switch” for selective stromal targeting and reprogramming.^10^ Our study, for the first time defined and scrutinized a set of miRNAs derived from CAFs, specifically in PDAC. Albeit the limitations of the study, these analyses support their tumor‐suppressive properties. Comprehensive and in‐depth experimental validation of the predicted miRNA‐RNA network construction and function is warranted. Thus, we forward a novel tumor‐suppressing mechanism of tumor‐restraining CAFs.

## CONFLICT OF INTEREST STATEMENT

The authors declare they have no conflicts of interest.

## FUNDING INFORMATION

National Institutes of Health, Award Number: 1U01DK108320; National Human Genome Research Institute of NIH, Grant Number: T32 HG008958

## Supporting information

Supporting Information

Supporting Information

Supporting Information
