# Supplementary material for: A peek at the other side of the coin: Tumor‐suppressor role of microRNAs expressed by pancreatic cancer‐associated fibroblasts
Source: Clin Transl Med. 2023 Aug 4;13(8):e1357. doi: 10.1002/ctm2.1357 (PMC10401917; doi:10.1002/ctm2.1357)
Supplement: Supplementary file 3 — Supporting Information [file CTM2-13-e1357-s001.docx]

Methods

1. Criteria and process for deCAF-miRNA selection. nanocount miRNA profiling identified 14 miRNAs that are highly expressed in CAFs in comparison to PDAC cells. We used the PubMed search engine from the National Center for Biotechnology information (NCBI) (<https://pubmed.ncbi.nlm.nih.gov/>) to gathering published references from the MEDLINE database. Search term used keywords of “miR-x [AND] cancer”, e.g. “miR-145 [AND] cancer”, to conduct the search for all cancer-related studies. Full paper of each article was read to ensure the mentioned mature sequence of -5p or -3p are in correspondent to our search goal. miRNAs with less than 20 reports were excluded from the study. microRNA Target Filter in Ingenuity Pathway Analysis (IPA) further defined 6 miRNAs are specifically involved in pancreatic adenocarcinoma signaling and thus defined as deCAF-miRNAs. All qualified articles were then assessed and sorted into two categories of tumor suppressive (TS-) or oncogenic (Onco-) based on their functions described from in vitro and / or in vivo studies.

Figure 1. Diagram of defining deCAF-miRNAs and their TS- or Onco- functions.

Figure 2. Diagram of

1. Bioinformatic Analysis. microRNA Target Filter in Ingenuity Pathway Analysis (IPA) was used for target gene identification. This includes using combined data sets of experimentally validated interactions from TarBase and miRecords, as well as predicted microRNA-mRNA interactions from TargetScan with high confidence filter. The sources for each predicted target gene were logged. The function and network of key targeted genes in pancreatic adenocarcinoma signaling was set as the focus for pathways and networks analysis. Target genes of the miRNAs were subjected to Gene ontology (GO) for curation of their biological process and molecular function. Kyoto encyclopedia of genes and genomes (KEGG) for pathway enrichment analysis was focused on pancreatic cancer. Only terms and pathways with adjusted p-value < 0.05 were considered significantly enriched and selected.
2. **Cell culture, miRNA Transfection and Cell viability test.** The human PDX-derived cell lines were cultured in DMEM/F12 media with 10% FBS and 1% antibiotic. Transfectio of miRNA mimics of miR-145 and miR-199a and non-specific control miRNA of C. Elegans (Cel-miR-39) were performed with 20 µl of preprepared RNAi duplex-Lipofectamine RNAiMAX (Invitrogen) complexes in 96-well of 2000 cells/well. Cell viability and cell proliferation was assessed at different time points using an alamarBlue assay (Thermo Scientific, Waltham, MA) and fluorescence intensity was measured using a Clariostar plate reader (BMG Labtech, Cary, NC). All statistical analysis was conducted using GraphPad Prism software (San Diego, CA, USA). The comparison of means between different groups was performed using non-parametric one-way ANOVA. Statistically significance was defined as a probability of p < 0.05.
